# Total hip arthroplasty through the direct anterior approach for sequelae of Legg–Calvé–Perthes disease

**DOI:** 10.1007/s00402-023-04791-4

**Published:** 2023-02-18

**Authors:** Julian Hasler, Andreas Flury, Armando Hoch, Frédéric Cornaz, Patrick O. Zingg, Stefan Rahm

**Affiliations:** Department of Orthopaedics, Balgrist University Hospital, University of Zurich, Forchstrasse 340, 8008 Zurich, Switzerland

**Keywords:** Perthes, DAA, THA, LCP Sequelae, Childhood hip disease

## Abstract

**Introduction:**

Due to multiplanar deformities of the hip, total hip arthroplasty (THA) for sequelae of Legg–Calvé–Perthes disease (LCPD) is often technically demanding. This study aimed to compare the clinical and radiographic outcomes of patients with sequelae of LCPD undergoing THA through the direct anterior approach (DAA) and non-anterior approaches to the hip.

**Methods:**

All patients with sequelae of LCPD who underwent primary THA between 2004 and 2018 (minimum follow-up: 2 years) were evaluated and separated into two groups: THA through the DAA (Group AA), or THA through non-anterior approaches to the hip (Group non-AA). Furthermore, a consecutive control group of patients undergoing unilateral THA through the DAA for primary hip osteoarthritis (Group CC) was retrospectively reviewed for comparison.

**Results:**

Group AA comprises 14 hips, group non-AA 17 hips and group CC 30 hips. Mean follow-up was 8.6 (± 5.2; 2–15), 9.0 (± 4.6; 3–17) and 8.1 (± 2.2; 5–12) years, respectively. At latest follow-up, Harris Hip Score was 90 (± 20; 26–100), 84 (± 15; 57–100), and 95 (± 9; 63–100) points, respectively. Overall, 6 patients treated for LCPD (each 3 patient in the AA and non-AA group) developed postoperative sciatic nerve palsy, of which only one was permanent. Complication-related revision rate at the latest follow-up was 15% in the AA-group and 25% in the non-AA group, respectively.

**Conclusion:**

THA through the DAA might be a credible option for the treatment of sequelae of LCPD with comparable complication rates and functional outcomes to non-anterior approaches.

## Introduction

Legg–Calvé–Perthes disease (LCPD) is characterized by osteonecrosis of the proximal femoral epiphysis during childhood caused by idiopathic disruption of blood flow to the femoral head [1–3]. While in some cases the osteonecrosis can resolve without alterations of the hip, multiplanar deformities of the proximal femur and the acetabulum occur in others [4–8]. Due to the resulting incongruence of the hip joint, patients with sequelae of LCPD are at high risk for early secondary osteoarthritis [4, 9] and often need to undergo total hip arthroplasty (THA) at young age [9, 10]. However, total hip replacement in these patients is often complicated by the altered anatomical conditions as well as previous surgical procedures undertaken during childhood, and the younger age puts patients at even higher risk for failure and further revision surgery [11, 12]. Currently, there’s only limited data in literature regarding the long-term outcome after THA in LCPD, yet outcomes in this group of patients seem to be less satisfactory with higher failure [13–15] and complication rates [16–18], compared to patients with primary osteoarthritis of the hip undergoing THA.

Using an internervous, intermuscular plane, the direct anterior approach (DAA) is gaining worldwide growing popularity and has demonstrated excellent functional and radiological outcomes in THA patients [19–21]. However, difficulties to expose the proximal femur [22, 23] and to perform acetabular augmentation might discourage surgeons from using the DAA in THA for sequelae of LCPD. Furthermore, performance of corrective osteotomies of the greater trochanter is hardly possible through the DAA, which might be a disadvantage in severe deformities with a high riding greater trochanter combined with minor leg length discrepancy.

Up to date, no literature exists concerning THA through the DAA for sequelae of LCPD. Therefore, the aim of the current study was to (1) review the peri- and postoperative complications as well as the clinical and radiographic outcomes of a group of patients undergoing THA through a minimally invasive DAA for sequelae of LCPD with a minimum follow-up of 2 years; (2) to compare the results with a group of patients undergoing THA for sequela of LCPD through non-anterior approaches to the hip; and (3) to compare the results to a control group of patients who underwent unilateral THA for primary osteoarthritis of the hip. We hypothesize that THA through the DAA for the treatment of sequelae of LCPD demonstrates comparable complication rates as well as clinical and radiographic outcomes compared to non-anterior approaches to the hip.

## Methods

This study was approved by the institutional review board and the ethical committee (ID 2020-00281). It was conducted entirely at the author’s institution and each patient provided written informed consent before participation. The inclusion criteria were adult patients, undergoing THA for secondary osteoarthritis related to LCPD, with a minimum follow-up of 2 years at the time of data collection and available preoperative radiographs of the hip. Patients without radiographically documented LCPD and a follow-up of less than 2 years were excluded from the study. All procedures were performed by a board-certified orthopaedic hip and pelvis surgeon, and the approach used was chosen by the treating surgeon according to the present deformity of the hip. Patients were separated into different groups: THA through the DAA (Group AA), or THA through either a trochanter osteotomy, lateral or posterior approach to the hip (Group non-AA). Additionally, a consecutive control group of patents aged between 30 and 65 years and treated with unilateral THA through the DAA for primary osteoarthritis of the hip with a minimum follow-up of 5 years (Group CC), was built for comparison.

### Patient characteristics

The medical records of all patients who underwent THA at our institution from January 2004 to June 2018 were retrospectively examined. From this database, a total of 45 patients (47 hips) were treated for sequelae of LCPD and met the inclusion criteria. Of these, 16 patients (16 hips) were lost to follow-up. Five patients died (mean follow-up: 14 months; complication recorded: One patient treated with THA through an anterolateral approach suffered a sciatic nerve palsy with a foot extension weakness (Medical research council (MRC) muscle scale 4) persisting to the latest follow-up one year postoperatively), 4 emigrated (mean follow-up: 10 months; no complications recorded), 6 could not be tracked through the local authority (mean follow-up: 36 months; no complication recorded), and 1 refused to participate.

**Fig. 1:**
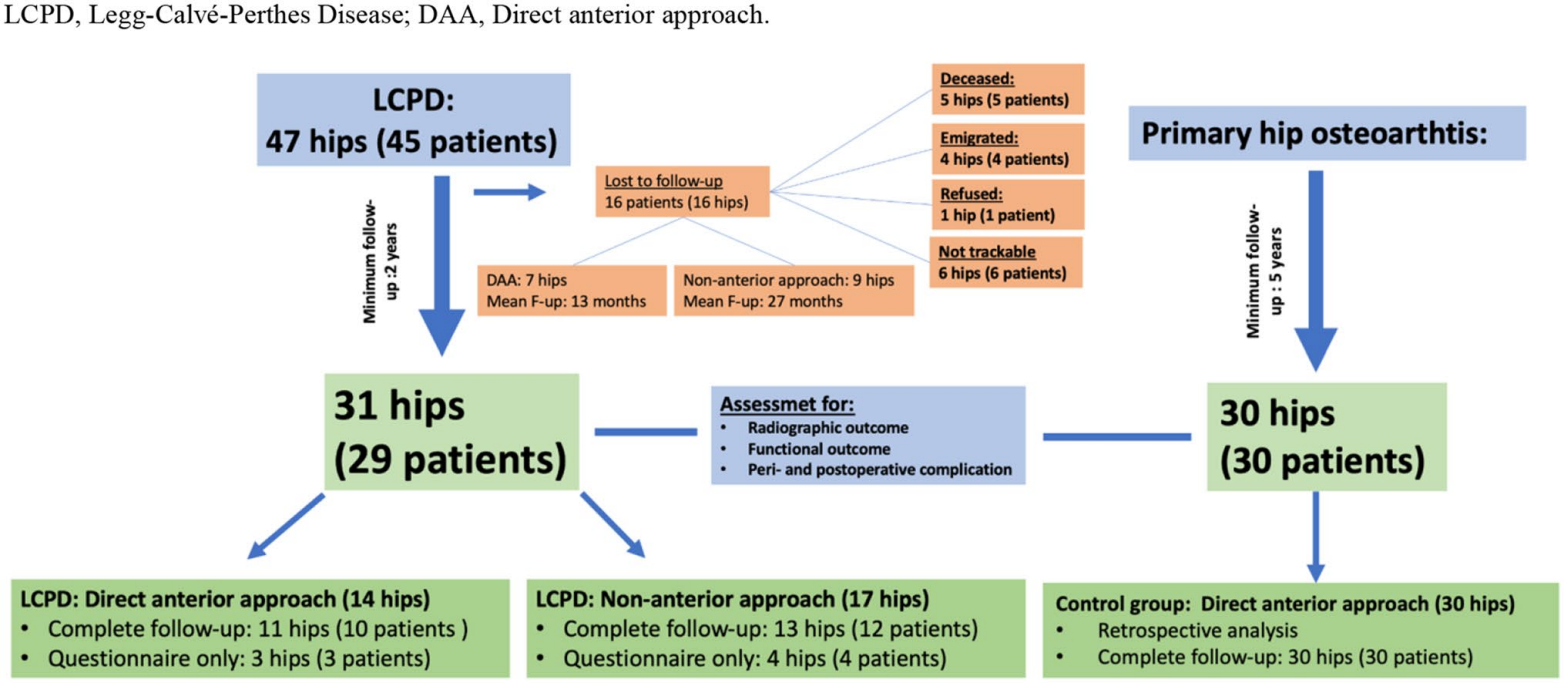
Flowchart of the study cohort and final study groups. *LCPD* Legg–Calvé–Perthes Disease, *DAA* Direct anterior approach

Finally, 13 patients (14 hips, 8 women and 6 men) with a mean age of 42 ± 10.5 years (range 27–69 years) and an average follow-up of 8.6 ± 5.2 years (range 2–15 years) treated through the DAA, and 16 patients (17 hips, 8 women and 9 men) with a mean age of 41 ± 12.8 years (24–67 years) and an average follow up of 9 ± 4.6 years (3–17 years) treated through a non-anterior approach to the hip were included in the study. Figure 1 shows a detailed patient enrollment flowchart with patients lost to follow-up.

#### Clinical Evaluation

Baseline characteristics including patient demographics and information regarding previous surgery of the affected hip were extracted from our clinical data system (Table 1). Medical records were reviewed for perioperative information (additional procedure performed with THA, operative time, estimated blood loss), peri- and postoperative complications and re-operations (analysis performed by J.H.).

**Table 1:** Patient Characteristics, previous procedures on the hip and functional outcome scores

	Group AA (*n* = 13 patients, 14 hips)	Group non-AA (*n* = 16 patients, 17 hips)	Control group (*n* = 30 patients and hips)	Significant differences (*P* value)
Age (years)	42.3 (10.5; 27–69)	41.5 (12.8; 24–67)	50.8 (6.9; 37–59)	Group CC > Group AA (*P* = 0.003*) Group CC > Group non-AA (*P* = 0.005*)
Gender				*P* = 0.750
Male (*n*)	6 (43%)	9 (53%)	17 (57%)	
Female (*n*)	8 (57%)	8 (47%)	13 (43%)	
ASA-Score				*P* = 0.524
1 (*n*)	8 (57%)	5 (29%)	11 (36%)	
2 (*n*)	5 (36%)	11 (65%)	17 (57%)	
3 (*n*)	1 (7%)	1 (6%)	2 (7%)	
4 (*n*)	0	0	0	
BMI (kg/m^2^)	27.6 (6.1; 19.3–34.2)	25.5 (4.7; 18.6–32.2)	26.2 (3.9; 19.1–35.2)	*P* = 0.580
Previous surgery proximal Femur	6 (43%)	12 (70%)	0	*P* = 0.160
Previous surgery Acetabulum	2 (14%)	8 (47%)	0	*P* = 0.068
Follow-up (years)	8.6 (5.2)	9 (4.6)	8.1 (2.2)	*P* = 0.874
Postoperative Harris Hip Score	90 (20.4; 26–100)	84 (15.2; 57–100)	95 (9.2; 63–100)	Group CC > Group non-AA (*P* = 0.004*) Group AA > Group non-AA (*P* = 0.024*)
Postoperative WOMAC Score	0.82 (1.05; 0–2.7)	2.03 (2.23; 0–8.5)	1.23 (2.10; 0–3.6)	*P* = 0.072
Subjective hip value (%)	78 (24.7; 0–100)	73 (21.5; 30–100)	87 (17.4; 40–100)	*P* = 0.059

The values were given as numbers and percentage or average value and standard deviation with range as appropriate

*ASA* American Society of Anestesiologist, *BMI* body mass index, *LLD* Leg length discrepancy, *NA* not available, *WOMAC* Western Ontario and McMaster Universities Osteoarthritis Index

*Statistically significant difference (*P* < 0.05)

Patients of the AA group and non-AA group were contacted by phone and invited to our outpatient clinic between June 2020 and November 2020. Clinical outcome was assessed with the Harris Hip Score (HHS) [24], the Western Ontario and McMaster Universities Osteoarthritis Index (WOMAC) [25] and the subjective hip value (SHV). 7 patients (7 hips) refused to show up for a follow-up visit at our outpatient clinic, but filled out questionnaires, including the WOMAC and SHV. Of all these patients, clinical information and radiographic images were available from the last follow-up.

### Radiographic measurements

**Fig. 2:**
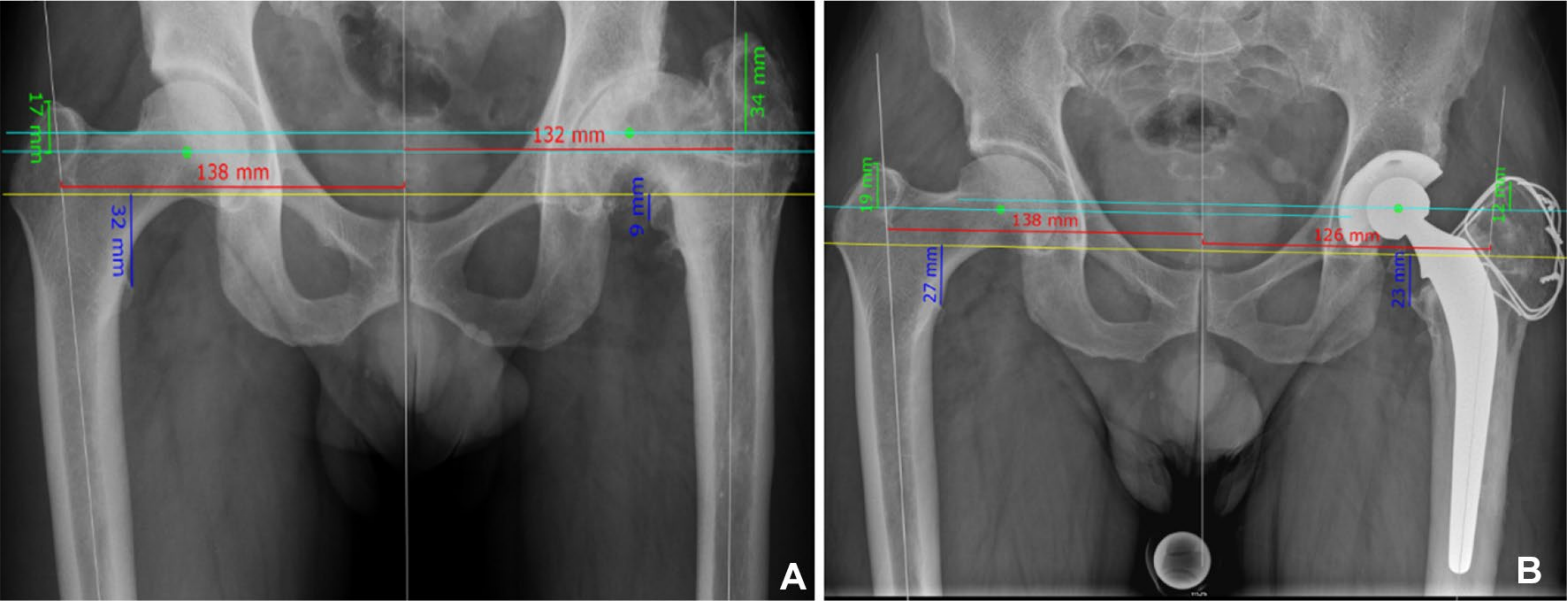
Preoperative (**A**) and postoperative (**B**) radiographic assessment of a patient with left-sided sequelae of LCPD. Yellow line: Inter-teardrop line. Red lines: Femoral offset (Sundsvall-method), measured at the height of the most lateral boarder of the greater trochanter as the horizontal distance between the midline of the pelvis and the femoral axis. Green lines: Height of the greater trochanter, measured as the distance between a line through the center of rotation of the hips perpendicular to the inter-teardrop line and the tip of the corresponding greater trochanter. Blue lines: Intraarticular leg length, defined as the perpendicular distance between the inter-teardrop line and the corresponding tip of the lesser trochanter

Standardized preoperative and postoperative anteroposterior pelvic and cross-table lateral radiographs of the hip were available for each patient included in the study. Additionally, a long-leg radiograph was obtained of every patient in the AA and non-AA group at the individual study visit, to objectively assess postoperative global leg length discrepancy. Radiographic evaluations were performed as a consensus read-out by J.H. (resident hip and pelvis surgery) and S.R. (board-certified orthopedic surgeon, senior consultant hip and pelvis surgery).On the preoperative radiographs, the following parameters were evaluated: Deformity of the hip according to the stulberg classification [4]; center of the femoral head using a best fit ellipse outlining the weight bearing area of the femoral head [26]; the lateral-center–edge angle (LCE); the acetabular index (AI); the height of the greater trochanter (HGT), measured as the distance from the center of hip rotation to the tip of the corresponding greater trochanter, on a line perpendicular to the inter-teardrop line [4, 26]; the intraarticular leg length discrepancy (LLD), measured from the tip of the lesser trochanter to the inter-teardrop line [27]; and the femoral offset difference between sides according to the Sundsvall-method [28] (Fig. 2).

On the postoperative radiograph, the HGT, the intraarticular LLD and the global offset were measured the same way as on preoperative radiographs. Leg lengthening assessment was calculated as the difference between the pre- and postoperative intraarticular LLD. To assess the acetabular cup position, the acetabular inclination was measured as the angle between the acetabular axis and the inter-teardrop line on the anteroposterior radiograph, and acetabular anteversion was measured in the cross-table lateral view as the angle between the acetabular axis and the coronal axis [29]. Additionally, in the AA and non-AA group, postoperative global LLD was measured on long-leg radiographs. For this, the distance of the hip center of rotation and the corresponding center of the talar dome was measured. This value was then corrected by the difference of vertical position of the corresponding hip center of rotation in relation to a line connection the uppermost points of the iliac crest.

### Statistical analysis

All parameters were tested using the Kolmogorov–Smirnov test for normality. When criteria for normality were met, continuous parameters were compared between groups using one-way ANOVA. Otherwise, a Kruskal–Wallis rank sum test was applied. In case of significance, pairwise comparisons using the *t* test or Wilcoxon rank sum test were established. Categorical variables were compared between groups using a Fisher exact Test. For significant parameters, a pairwise Fisher exact Test was used for pairwise comparison. *P* values < 0.05 are considered statistically significant. All statistical analyses were performed using statistical software R (R Studio, Boston, Massachusetts, USA).

## Results

### Patient characteristics

A summary of patient characteristics is provided in Table 1. No difference between LCPD groups was observed for age, gender, BMI, the patients`physical status according to the American Society of Anesthesiologists (ASA) and previous surgeries of the femur or acetabulum. The control group was a mean of 8 years older compared to the LCPD groups.

### Perioperative parameters

Table 2 provides further information on perioperative parameters in the different subgroups. In the non-AA group, 11 procedures were performed through a trochanteric osteotomy, 2 procedures through a lateral approach, and 4 procedures through a posterior approach to the hip. In 10 patients, a concomitant trochanteric distalization was performed. Concomitant removal of osteosynthesis material was performed in 1 patient in the AA group, and in 2 patients in the non-AA group. Simultaneous acetabular augmentation was performed in 1 patient in the AA group, and in 5 patients in the non-AA group.

**Table 2:** Perioperative parameters and additional procedures performed with THA

	Group AA (*n* = 13 patients, 14 hips)	Group non-AA (*n* = 16 patients, 17 hips)	Control group (*n* = 30 patients and hips)	Significant differences (*P* value)
Operation duration (min)	123 (33; 70–180)	175 (56; 85–270)	101 (28; 70–140)	Group AA > Group CC (*P* = 0.041*) Group non-AA > Group AA (*P* = 0.011*) Group non-AA > Group CC (*P* < 0.001*)
Intraoperative blood loss (ml)	573 (194; 300–900)	587 (370; 300–1500)	488 (263; 200–1500)	*P* = 0.302
Duration of hospital stay (days)	5.5 (1.2; 4–8)	7.5 (1.8; 5–13)	5.4 (1.7; 3–10)	Group non-AA > Group AA (*P* = 0.001*) Group non-AA > Group CC (*P* < 0.001*)
Additional EMO	1 (7%)	2 (12%)	0	NA
Acetabular augmentation	1 (7%)	5 (29%)	0	NA
Trochanteric osteotomy	0	11 (65%)	0	NA
Trochanteric distalization		10 (59%)		

The values were given as numbers and percentage or average value and standard deviation with range as appropriate

*EMO* Extraction of osteosynthesis material, *THA* Total hip arthroplasty, *NA* not available

*Statistically significant difference (*P* < 0.05)

### Functional outcome

At the latest follow-up, good to excellent results were observed in all subgroups. Pairwise comparison revealed a significantly lower HHS in the non-DAA group compared to the DAA group (*P* = 0.024) and the CC group (*P* = 0.004). (Table 1).

### Radiographic findings

Table 3 provides a summary of the radiographic findings and significant differences between groups. No significant difference was found for residual femoral head deformity and joint congruence according to the Stulberg classification between the LCPD groups (*P* = 0.593). Nonetheless, the non-AA group demonstrated more dysplastic acetabula with a significantly smaller LCE of 17 ± 9.7° and higher AI of 18 ± 10.4° compared to the AA group with a mean LCE of 24 ± 10.7° (*P* = 0.020) and mean AI of 12 ± 5.5° (*P* = 0.016). Furthermore, the non-AA group demonstrated a significantly higher preoperative position of the GT in relation to the femoral head compared to the AA group (25 ± 14.6 mm vs. 12 ± 8.8 mm, *P* < 0.001). Postoperatively, the mean trochanteric height was successfully normalized in all groups (*P* = 0.236). The postoperative global LLD (available for 10 hips in the AA group and 14 hips in the non-AA group) was similar in the AA and non-AA group with 7.2 ± 6.1 mm and 6.5 ± 7.3 mm, respectively (*P* = 0.636). Additionally, the mean postoperative intraarticular LLD was similar between groups with 1.9 ± 8.1 mm in the AA group and -3.1 ± 6.1 mm in the non-AA group, respectively.

**Table 3:** Pre- and postoperative radiographic analysis

	Group AA (*n* = 13 patients, 14 hips)	Group non-AA (*n* = 16 patients, 17 hips)	Control group (*n* = 30 patients and hips)	Significant differences (*P* value)
Stulberg-classification			NA	*P* = 0.593
1	2 (14%)	0		
2	0	2 (12%)		
3	6 (42%)	9 (53%)		
4	3 (22%)	6 (35%)		
5	3 (22%)	0		
Preoperative LCE (°)	24 (10.7; 1 to 35.5)	17 (9.7; 4.8 to 29.8)	28 (5.1; 22.1 to 38.8)	Group non-AA < Group AA (*P* = 0.020) Group 2 < Group 3 (*P* < 0.001*)
Preoperative AI (°)	12 (5.5; 5.1 to 24.5)	18 (10.4; − 1.7 to 34.5)	9 (2.3; 4.7 to 13.8)	Group non-AA > Group AA (*P* = 0.016*) Group non-AA > Group CC (*P* < 0.001*)
Preoperative height of the GT (mm)	12 (8.8; − 6 to 23)	25 (14.6; − 1 to 49)	1 (5.0; − 9 to 10)	Group AA > Group CC (*P* < 0.001*) Group non-AA > Group AA (*P* < 0.001*) Group non-AA > Group CC (*P* < 0.001*)
Postoperative height of the GT (mm)	1 (7.2; − 15 to 9)	3 (10.7; − 12 to 22)	− 2 (7.4; − 17 to 9)	*P* = 0.236
Correction of GT	− 11.7 (7.8; − 27 to − 3)	− 22.1 (12.1; − 41 to − 2)	− 3.3 (5.7; − 17 to 5)	Group AA > Group CC (*P* = 0.002*) Group non-AA > Group AA (*P* = 0.001*) Group non-AA > Group CC (*P* < 0.001*)
Preoperative intraarticular LLD (mm)	− 9 (11.0; − 31 to 7)	− 13.6 (11.9; − 36 to 4)	− 2.5 (2.5; − 7 to 3)	Group CC < Group AA (*P* = 0.038*) Group CC < Group non-AA (*P* = 0.003*)
Postoperative intraarticular LLD (mm)	1.9 (8.1; − 10 to 17)	− 3.1 (6.1; − 20 to 3))	1.6 (3.2; − 4 to 8)	Group non-AA > Group CC (*P* = 0.005)
Leg lengthening	10.9 (7.8)	12.3 (11.5)	4.1 (3.3)	Group AA > Group CC (*P* = 0.006*)
Number of hips lengthened	13 (93%)	15 (8%)	28 (93%)	Group non-AA > Group CC (*P* < 0.001*)
Postoperative global LLD (mm)	7.2 (6.1; − 18 to 18)	6.5 (7.3; − 27 to 10)	NA	*P* = 0.636
Preoperative femoral offset difference (mm)	6 (4.0; − 14 to 9)	9 (8.5; − 18 to 32)	2 (2.5; − 5 to 12)	*P* = 0.056
Postoperative femoral offset difference (mm)	7 (3.4; − 13 to 8)	11 (7.1; − 24 to 13)	3 (3.3; − 7 to 16)	Group CC < Group AA (*P* = 0.004*) Group CC < Group non-AA (*P* < 0.001*)
Acetabular inclination (°)	43 (5.0; 38 to 53)	39 (6.4; 26 to 49)	41 (4.8; 28 to 49)	*P* = 0.466
Acetabular anteversion (°)	29 (2.7; 22 to 34)	27 (7.5; 7 to 30)	27 (5.4; 17 to 35)	*P* = 0.387

The values were given as average value and standard deviation as appropriate

*AI* Acetabular Index, *LLD* Leg length discrepancy, *LCE* lateral center edge angle, *GT* greater trochanter, *NA* not available

^*^Statistically significant difference (*P* < 0.05)

### Complication and re-operation rate

A summary of the complication and reoperation rates is listed in Table 4. At the latest follow-up, 4 patients in the AA group and 7 patients in the non-AA group sustained a total of 5 and 10 complications, respectively. Complication-related revision rate at the latest follow-up was 15% in the AA-group and 25% in the non-AA group.

**Table 4:** Summary of Complications

	Group AA (*n* = 13 patients, 14 hips)	Group non-AA (*n* = 16 patients, 17 hips)
Number of patients with complications	4 (31%)	7 (44%)
Number of patients with reoperation	2 (15%)	6 (38%)
Due to complications	2 (15%)	4 (25%)
Removal of osteosynthesis material	0	2 (13%)
Periprosthetic femur fractures	1	1
Intraoperative	1	0
Late postoperative	0	1
Hematoma needing revision	0	0
Non-Union of greater trochanter needing revision	0	3 (18%)
Infection	1	1
Early	1	0
Late	0	1
Sciatic nerve injury	3	3
Transient	2	3
Persistent	1	0
Dislocation	0	1
Closed reduction	0	1
Open reduction	0	0
Aseptic loosening	0	1
Heterotopic ossification needing revision	0	0

The values were given as numbers and percentage

Intraoperative periprosthetic fracture occurred in 1 patient in the AA group, which was successfully treated with cerclage wires. In the non-AA group, 3 patients underwent revision surgery due to non-union and secondary dislocation of the greater trochanter after trochanteric osteotomy. At the latest follow-up, only one patient showed radiographic union. One patient with persistent non-union underwent one-staged exchange arthroplasty with resection of the remaining trochanter due to a late periprosthetic joint infection 7 years after the primary procedure. The other patients with persistent non-union underwent removal of the cerclage wires without refixation due to symptomatic bursitis. Three patients in the AA group developed postoperative sciatic nerve palsy: One resolved spontaneously, and one resolved after revision THA with shortening of the operated leg 2 years after the primary procedure. In one patient, the injury was permanent with a foot extensor weakness (MRC 4). Likewise, in the non-AA group, three patients developed sciatic nerve palsy, all of which resolved spontaneously. The patients in the AA group were lengthened by 7, 14 and 21 mm (mean 14 mm), compared to a mean of 11 mm in the patients who did not sustain a neurologic injury. Likewise, the patients in the non-AA group were lengthened by 11, 19 and 20 mm (mean 17 mm), compared to a mean of 12 mm in the patients who did not sustain a neurologic injury in the same group.

## Discussion

Sequelae of LCPD is a rare condition, responsible for less than 0.6% of all primary THA [30], with currently only limited data available in literature. This study reports the clinical and radiographic outcomes of 13 patients (14 hips) undergoing THA for LCPD through a minimally invasive DAA. The most important finding of the current study was that the functional outcome was comparable in the anterior approach group and the control group. Furthermore, the anterior approach group showed higher HHS and a lower complication rate compared to the non-anterior approach group at the latest follow-up.

Various studies reported significant functional outcome improvement in patients undergoing THA for LCPD. In a systematic review including 245 patients undergoing THA for LCPD with a mean follow-up of 8.4 years, Hanna et al. reported an average improvement of the HHS of 38 points to 89.7 postoperatively [15]. Baghdadi et al. reported on 95 patients with sequelae of LCPD undergoing THA either through a transtrochanteric, anterolateral or posterior approach to the hip. In their study, the HHS improved from 56 to 88 points at the latest follow-up [16]. Comparably, the current study demonstrated satisfactory functional outcome scores with a mean HHS and WOMAC-score of 87 ± 17.7 and 1.53 ± 1.91 points for all patients undergoing THA for LCPD. Furthermore, subgroup analysis revealed a significantly higher HHS at the latest follow-up in the AA group and CC group compared to the non-AA group. This might partially be explained by the more pronounced preexisting hip deformities found in the non-AA group compared to the other subgroups (Table 3), resulting in more invasive surgery, sometimes even with corrective trochanteric osteotomies, necessary to restore symmetric anatomical conditions.

One main challenge in patients undergoing THA for LCPD is the restoration of normal hip biomechanics, especially considering the generally young patient age and the high rate of unilateral involvement. Luo et al. reported on 71 patients who underwent cementless THA for LCPD through a posterolateral approach. While they reported a mean preoperative LLD of 24.3 ± 7.8 mm (range 8–36 mm), they achieved almost equal leg length with a mean postoperative LLD of 2.4 ± 2.8 mm (range − 2 to 9 mm) [31]. Traina et al. investigated 32 hips undergoing THA for LCPD through a direct lateral approach. They reported a mean preoperative LLD of 12 mm (range 0–30 mm), which was only partially corrected with a mean postoperative LLD of 9 mm (range 0–26 mm)[10]. In the presented study, mean preoperative LLD was − 11.5 ± 11.6 mm for all patients undergoing THA for LCPD, with no significant difference (*P* = 0.233) between the AA group (− 9 ± 11.0 mm) and the non-AA group (− 13.6 ± 11.9 mm). Similarly to the study of Traina et al., there was a high range of postoperative LLD in both subgroups (mean LLD of 1.0 ± 8.1 mm (range 17 to − 10 mm) in the AA group, and − 3.1 ± 6.1 (range 3 to − 20 mm) in the non-AA group), thus emphasizing the difficulty to achieve leg length equality in these patients, regardless of the approach chosen for THA.

Seufert et al. reported on the trochanteric height in 14 hips undergoing THA trough a posterior approach for LCPD. While they measured a mean trochanteric height of 23.6 mm preoperatively, they were able to achieve a more anatomic relationship with a mean trochanteric height of 6.8 mm postoperatively [26]. Similarly, in the current study, restoration of a normal anatomic relationship between the greater trochanter and the center of hip rotation was successfully achieved with a mean postoperative trochanteric height of 1.5 ± 9.2 mm. However, subgroup analysis revealed a significant difference of the preoperative trochanteric height between the AA group and the non-AA group (12 ± 8.8 mm vs. 25 ± 14.6 mm, *P* < 0.001). Thus, distalization of the greater trochanter was much greater in the non-AA group (− 22.1 ± 12.1 mm) compared to the AA group (− 11.7 ± 7.8 mm), which was only possible due to a trochanteric osteotomy performed in 11 patients in the non-AA group. Without corrective osteotomy of the trochanter and equal leg lengthening in these 11 patients, the mean postoperative trochanteric height would have been 13,3 ± 9.4 mm, compared to 5.7 ± 11 mm with concomitant distalization of the greater trochanter. Thus, simultaneous correction of the intraarticular leg length and the position of the greater trochanter would not have been possible without a trochanter osteotomy. Hence, if the difference in height of the greater trochanter largely exceeds the intraarticular leg length discrepancy, the DAA should not be used, since normalization of the anatomical conditions in such cases requires a trochanteric osteotomy.

The current study demonstrates a significantly higher complication and revision rate in patients undergoing THA for LCPD, compared to the CC group. Postoperative sciatic nerve palsy is a frequently observed complication following THA for LCPD. Al-Khateeb et al. reported two (13%) permanent sciatic nerve palsy in their cohort of 15 patients undergoing THA for LCPD [32]. Similarly, Traina et al. reported two (7%) permanent sciatic nerve palsy in 27 patients undergoing THA for LCPD [10]. In the presented study, three patients in the AA group and three patients in the non-AA group developed postoperative sciatic nerve palsy. However, of these, four resolved spontaneously and one resolved after revision THA with shortening of the operated leg 2 years after the primary procedure, while only one patient in the AA group showed permanent foot extensor weakness (MRC 4). Thus, the rate of permanent sciatic nerve palsy in the presented cohort was 3%, which is comparable to the reported literature.

Another frequently observed complication associated with THA for sequelae of LCPD is intraoperative femoral fracture. In a systematic review, Hanna et al. reported intraoperative femoral fracture to occur in 11% of patients undergoing THA for LCPD, all of which happened with the use of standard femoral stems [15]. Similarly, Sansanovicz et al. found a higher risk of intraoperative femoral fractures in patients undergoing THA for LCPD compared to a control group of patients undergoing THA for primary osteoarthritis [18]. Conversely, Seufert et al. found no intraoperative periprosthetic fractures in a cohort of 28 patients undergoing THA for LCPD using a modular type femoral component [26]. Interestingly, intraoperative periprosthetic fracture occurred in only one patient in presented study, although only monobloc implants have been used.

The current study should be interpreted in light of its potential limitations. The study only represents a relatively small cohort with only a limited number of patients available in each subgroup. However, it should be considered that less than 0.6% of all primary THA are performed due to sequelae of LCPD, thus collection of a larger cohort is difficult to achieve. Due to the long follow-up of many of the included patients, no preoperative information such as the HHS, WOMAC or SHV are available, as these were not standardly collected during the time of preoperative assessment. Therefore, no statement can be made regarding the improvement of the scores during follow-up visits. Additionally, due to the young age of patients undergoing THA for sequelae of LCPD, generation of an age- and gender-matched control group of patients undergoing unilateral THA for primary osteoarthritis of the hip was not possible, which complicates comparison of the different subgroups. Furthermore, the approach used for THA was mainly dictated by the existing hip deformity of the patients and chosen by the treating surgeon. As mentioned above, restoration of normal anatomic conditions in patients with a high riding trochanter and only minor leg length discrepancy requires a corrective osteotomy of the greater trochanter. Thus, retrospective comparison of the subgroups was complicated due to the generally more pronounced deformities in the non-AA group compared to other subgroups.

In conclusion, THA through the DAA might be a credible option for the treatment of sequelae of LCPD with comparable complication rates and functional outcomes to non-anterior approaches to the hip.

## Data Availability

The datasets generated and analyzed during the current study are available from the corresponding author on reasonable request.
